# Supplementary material for: Investigating the Role of BATF3 in Grass Carp (Ctenopharyngodon idella) Immune Modulation: A Fundamental Functional Analysis
Source: Int J Mol Sci. 2019 Apr 4;20(7):1687. doi: 10.3390/ijms20071687 (PMC6479329; doi:10.3390/ijms20071687)
Supplement: Supplementary file 1 [file ijms-20-01687-s001.pdf]

```
   1 cgcgatgggggcactgtagcacgttgactgacagtttaaaaatggataactttgcgctaattaacacagcctcgtaatttgctttaattt
   1                                                           M  S  L  F  N  A  T  S  N  F  T
  91 tctatctagtttagtggacgtaaatcttcattgtgcgcgagctctcctgcacctgccATGTCACTTTTCAATGCGACAAGTAACTTTACT
  12 R  S  D  A  P  A  L  R  L  F  R  K  S  E  S  S  D  D  D  E  K  R  L  K  R  R  E  K  N  R
 181 CGAAGTGATGCTCCAGCTTTACGGTTGTTTCGAAAGAGTGAGAGCTCTGATGATGATGAGAAAAGGTTGAAAAGAAGAGAAAAGAACCGA
  42 V  A  A  Q  R  S  R  K  R  Q  T  Q  R  A  D  E  L  H  E  A  Y  E  C  L  E  Q  E  N  S  L
 271 GTTGCTGCCCAGAGAAGCCGCAAAAGACAAACCCAGAGAGCTGACGAGTTGCACGAGGCGTATGAGTGTCTGGAACAGGAGAACAGCCTG
  72 L  R  K  E  V  Q  L  L  I  E  E  Q  Q  R  L  T  D  A  L  K  A  H  E  P  L  C  P  V  L  N
 361 CTGAGGAAGGAAGTCCAGCTTTTGATAGAGGAACAGCAACGCTTAACAGATGCCCTCAAAGCCCATGAGCCTTTGTGCCCTGTCTTGAAC
 102 C  G  M  T  S  T  T  R  S  T  G  T  V  P  Q  D  I  H  I  *
 451 TGTGGTATGACCTCAACAACAAGGTCCACAGGCACAGTGCCACAAGACATTCACATCTGAtgctcatgatgtcggaatttccatttaagg
 541 ataaattaagctaattatctttaatacaagtgattcgtgcatactagcggcatgcagtttttatgctttttattctaaagtattaacccg
 631 tgagttcagtatccgttaacatgcagttgaattaattattcatgctcgaactttctcttttgcgtttttgacaggtttcgttcagaatac
 721 agaatctctagtcatatgttacttttcccagggccatgcctcacgtgaatacttccacttttggtatttactgagatagagtaaataacc
 811 actaactaaaccatctgttataccacagttagtggactgtgaatgaacaacatgcgagaatggacttctgctttatgagagcctatccga
 901 ccaaaatactaaaggttagtttcaaaactctgttacagccgctttgttatggctgcactattacaaataaccacaaatggcagtgttact
 991 attctgtcttgcttggtcatgttttgctgtgggtatgtattgtaagtagaagaataattttattttaataaaaaggaattactctgttta
1081 tacactctgtgacgcagatgcagattaaaatccagtattacttcaattgtaatagtgatacaatttctattaaaatgaggtcaccaaatc
1171 agaaaaaaaaaaaaaaaaaaaaaaaaa
```
